# Role of Polypyrimidine Tract Binding Protein in Mediating Internal Initiation of Translation of Interferon Regulatory Factor 2 RNA

**DOI:** 10.1371/journal.pone.0007049

**Published:** 2009-09-16

**Authors:** Debojyoti Dhar, Musturi Venkataramana, Anand Ponnuswamy, Saumitra Das

**Affiliations:** Department of Microbiology and Cell Biology, Indian Institute of Science, Bangalore, Karnataka, India; University of Hyderabad, India

## Abstract

**Background:**

Earlier we have reported translational control of interferon regulatory factor 2 (IRF2) by internal initiation (Dhar *et al*, Nucleic Acids Res, 2007). The results implied possible role of IRF2 in controlling the intricate balance of cellular gene expression under stress conditions in general. Here we have investigated the secondary structure of the Internal Ribosome Entry Site of IRF2 RNA and demonstrated the role of PTB protein in ribosome assembly to facilitate internal initiation.

**Methodology/Principal Findings:**

We have probed the putative secondary structure of the IRF2 5′UTR RNA using various enzymatic and chemical modification agents to constrain the secondary structure predicted from RNA folding algorithm Mfold. The IRES activity was found to be influenced by the interaction of *trans-*acting factor, polypyrimidine tract binding protein (PTB). Deletion of 25 nts from the 3′terminus of the 5′untranslated region resulted in reduced binding with PTB protein and also showed significant decrease in IRES activity compared to the wild type. We have also demonstrated putative contact points of PTB on the IRF2–5′UTR using primer extension inhibition assay. Majority of the PTB toe-prints were found to be restricted to the 3′end of the IRES. Additionally, Circular Dichroism (CD) spectra analysis suggested change in the conformation of the RNA upon PTB binding. Further, binding studies using S10 extract from HeLa cells, partially silenced for PTB gene expression, resulted in reduced binding by other *trans*-acting factors. Finally, we have demonstrated that addition of recombinant PTB enhances ribosome assembly on IRF2 IRES suggesting possible role of PTB in mediating internal initiation of translation of IRF2 RNA.

**Conclusion/Significance:**

It appears that PTB binding to multiple sites within IRF2 5′UTR leads to a conformational change in the RNA that facilitate binding of other *trans*-acting factors to mediate internal initiation of translation.

## Introduction

Initiation of translation of the majority of the eukaryotic cellular mRNAs is mediated by the cap-dependent mode of translation which results from binding of ribosomes to the m^7^G cap at the 5′ end of the mRNA followed by linear scanning to the initiation codon [1]. However many viral and a small number of cellular RNAs have been shown to initiate translation by ‘Internal Ribosome Entry Site (IRES)’ mechanism in a 5′cap-independent manner [2]. Internal initiation is thought to facilitate translation of certain mRNAs under conditions when cap-dependent translation is less efficient, such as under heat shock, amino- acid starvation etc [3], [4]. Recently we have shown that the 5′UTR of the interferon regulatory factor 2 (IRF2) mRNA contains an IRES element. Its activity does not seem to be affected under various stress conditions such as ER stress, coxsackievirus 2A protease treatment etc. [5].

Interferon regulatory factors are DNA-binding proteins, which are known to control interferon (IFN) gene expression. IRF1 function as an activator of IFN and IFN-inducible genes, whereas IRF2 act as repressor of IRF1 action [6]. Interestingly, IRF2 has also been implicated to stimulate certain genes under various conditions, such as vascular cell adhesion molecule (VCAM), histone H4 genes etc [7], [8]. The IRES activity of IRF2 gene ensures basal levels of the IRF2 protein, which might have a role in cellular response to various conditions of stress. The exact mechanism of the cellular IRES activity is still not completely understood. But it is believed that certain IRES *trans* acting factors or ITAFs might play an important role in the function [9]. Several ITAFs, such as La, PTB, unr, hnRNPC have been shown to influence the activity of various cellular IRES [10]–[12]. Earlier we have shown that Polypyrimidine tract binding protein (PTB) interacts with the 5′ UTR of the RNA and was found to be essential for its activity [5]. PTB has been shown to act as RNA chaperone and facilitate Apaf1-IRES structure and influence the efficiency of its translation initiation [13]. It has also been reported to positively regulate the IRES mediated translation of HIF-1α, p27kip1 [14], [15].

We have partially mapped the secondary structure of the IRF2 5′UTR using various enzymatic and chemical modification probes. The data validates the predicted MFOLD structure of the 5′UTR RNA. To map the putative contact points of the protein on the IRF2 5′ UTR we have performed primer extension inhibition assay. PTB was found to have multiple contact points on the IRF2 RNA, but mostly at the 3′end of the 5′UTR. Since many contact points were found on the 3′ end of the RNA, a deletion mutant was generated, where 25 nt from 3′end was deleted. UV cross-linking and competition experiments showed a reduced binding of the purified PTB on the mutant RNA as compared to the wild type RNA. The above data was supported by Circular Dichroism (CD) spectra analysis of the RNA, which also suggested a change in the conformation of IRF2 5′ UTR in presence of PTB binding. Proteins from whole cell extracts showed that full length IRF2 IRES interacted predominantly with a 57 kDa (PTB) protein, compared to the mutant IRF2. Further PTB silencing experiments showed reduced PTB binding along with significant reduction in binding of other cellular *trans*-acting factors. Ribosome binding analysis also indicated that supplementation of purified PTB enhanced the formation of translation initiation complex. This led us to hypothesize that PTB interaction with the IRF2 IRES might trigger a change in the conformation that helps in the recruitment of other hitherto unknown *trans* acting factors and ribosomes, thus facilitating translation initiation.

## Results

### Probing the secondary structure of the IRF2 IRES

#### DMS modification

Earlier we have shown that the putative secondary structure of IRF2 5′UTR RNA (predicted by using the MFOLD program) [5], [16]. Here we have probed the secondary structure of IRF2 IRES RNA by monitoring the accessibility of nucleotides to chemical modification (DMS) and enzymatic digestions. Dimethylsulphate (DMS) reacts with N-1 position of adenine residues and to N-3 of cytosine residues in the single stranded regions [18]. Base pairing inhibits chemical modifications at these positions. Since chemically modified residues are unable to base pair with the complementary deoxynucleotides, modification results in the inhibition of the primer extension by the RT one nucleotide before the point of modification.

**Figure 1:**
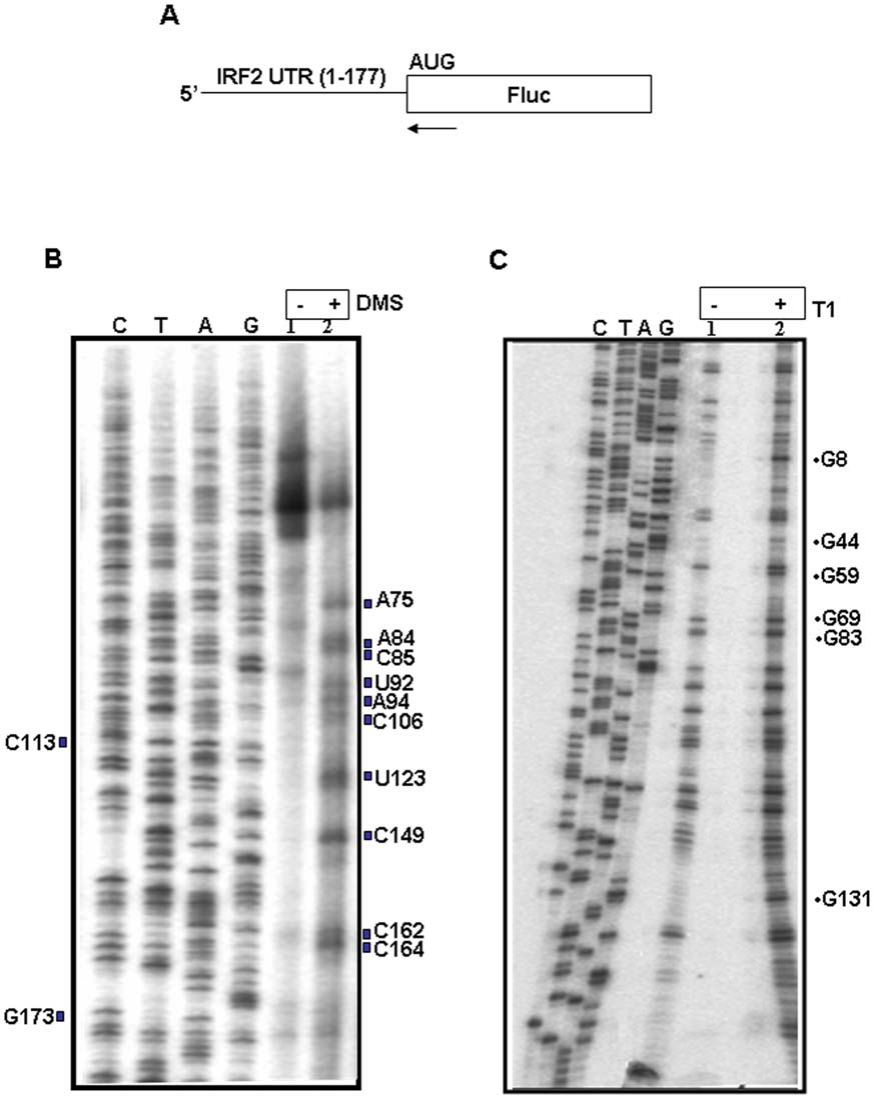
Probing of secondary structure of IRF2 UTR RNA with DMS and RNase T1: (A) A schematic representation of IRF2-Luc monocistronic construct used for generating *in vitro* transcripts by using T7 RNA polymerase. A 20 nucleotide long -^32^P labeled reverse primer was used for reverse transcription indicated by an arrow at the 5′ end of luciferase. (B) IRF2 5′ UTR RNA was incubated with (lane 2) or without (lane 1) dimethyl sulphate (DMS). The unmodified and modified RNAs were reverse transcribed and cDNAs were resolved parallel with a sequencing reaction performed with the same end labeled primer. Modified nucleotides are indicated on the right of the panel. (C) IRF2 5′ UTR RNA was incubated with or without of 1.0 unit of RNase T1 and the undigested and digested RNAs were reverse transcribed and cDNAs were resolved in parallel with a sequencing reaction performed with the same end labeled primer. Putative cleavage points are indicated by arrows on the right of the panel.

IRF2 monocistronic RNA was generated from the corresponding DNA construct (Fig. 1A). The RNA was treated with DMS and followed by primer extension using AMV-RT. The products were then analyzed on 8% polyacrylamide-8M urea denaturing gel in conjunction with corresponding DNA sequencing ladder to allow the identification of modified nucleotides. Several RT pauses were observed, which include A75, A84, C85, U92, A94, C106, U123, C149, C162, C164 (Fig. 1B). The data suggests that the IRF2 IRES is highly structured as very few nucleotides were modified by DMS. The modified nucleotides were mostly found in the single stranded region of the RNA which validates the MFOLD prediction.

### RNase T1 digestion

Further, RNase T1 digestion was carried out following primer extension inhibition assay protocol described in material and methods. *in vitro* transcribed IRF2 UTR RNA was incubated with RNase T1 followed by extension using AMV-RT (Promega). The products were analyzed on 8% polyacrylamide-8M urea denaturing gel. For precise mapping of the contact points, a DNA sequencing reaction with the same end labeled primer was run alongside (labeled as C, T, A, G). Since IRF2 5′UTR folds into a fairly stable secondary structure, only few specific RT pauses were observed at G8, G44, G59, G69, G83 and G131 (Fig. 1C). The putative pauses were mapped on the predicted secondary structure (MFOLD) of IRF2 IRES RNA.

### RNase VI digestion

**Figure 2:**
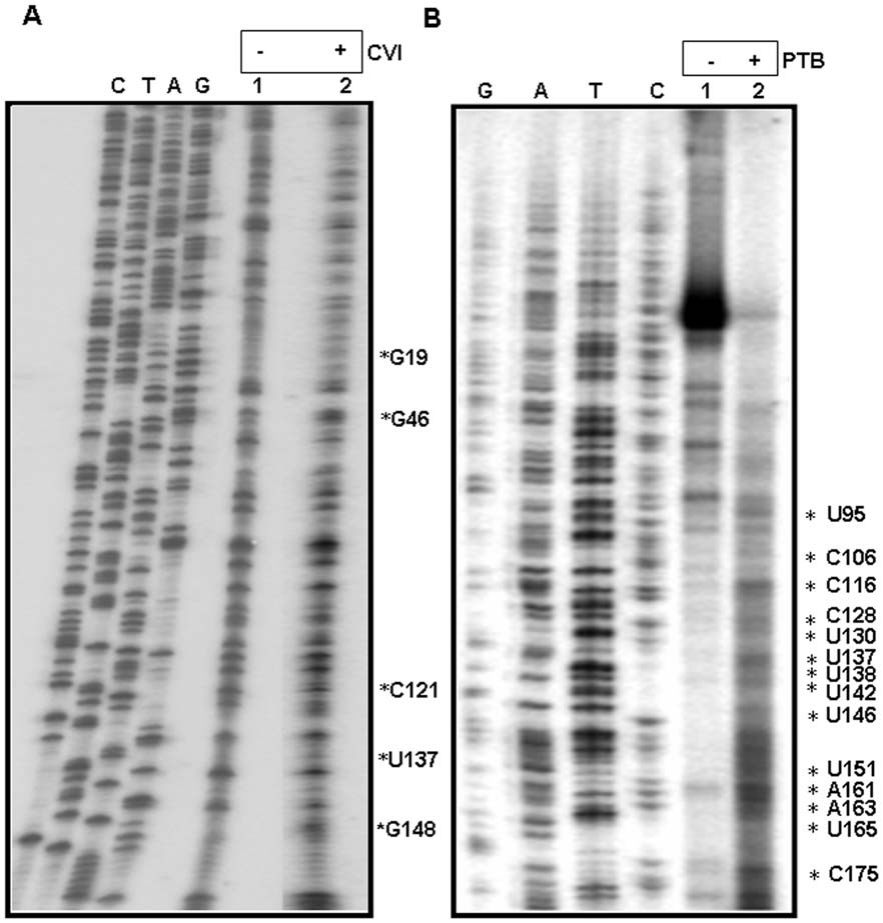
RNase VI digestion and Toe-printing of PTB on IRF2 UTR RNA: (A) IRF2 5′ UTR RNA was incubated with (lane 2) or without (lane 1) of 0.01 units of RNase V1. The unmodified and modified RNAs were reverse transcribed and cDNAs were resolved parallel with a sequencing reaction performed with the same end labeled primer. Cleaved nucleotides are indicated on the right of the panel. (B) For toe-printing, IRF2 wt 5′ UTR RNA was incubated in absence or presence of purified recombinant PTB protein. The RNAs in the Ribonucleoprotein complexes were reverse transcribed and the cDNAs were resolved in 8M urea 8% PAGE in parallel with a sequencing ladder corresponding to IRF2 5′UTR RNA obtained by using the same end labeled primer. The cDNA products terminated at the sites due to protein binding is indicated on the right.

In addition, IRF2 IRES RNA was further probed by RNase VI cleavage. RNase VI is from cobra venom which cleaves double stranded or other base paired regions [17]. For this purpose, the IRF2 RNA was incubated with RNase VI and extracted with phenol:chloroform. The precipitated RNA components were reverse transcribed using a radiolabeled 3′ primer and extended by AMV-RT. The products were then analyzed on 8% polyacrylamide-8M urea denaturing gel in conjunction with corresponding DNA sequencing ladder to allow identification of modified residues (Fig. 2A). The data supported highly structured IRF2 UTR as observed in above experiments and showed very few RNase V1 modified nucleotides. These pauses include (G19, G46, C121, U137 and G148) (Fig. 2A). These nucleotides are expected to be in double-stranded region of the RNA and thus validate the predicted MFOLD structure.

### Analysis of PTB binding site by Toe printing

The binding region of PTB on IRF2 UTR was further analyzed by Toe-printing. The unlabeled RNA was incubated with PTB and primer extension inhibition assay was carried out as described earlier. The intensity of few RT pauses was increased in presence of PTB suggesting possible contact points. The pauses corresponds to U95, C106, C116, C128, U130, U137, U138, U142, U146, U151, A161, A163, U165, C175 (Fig. 2B).

**Figure 3:**
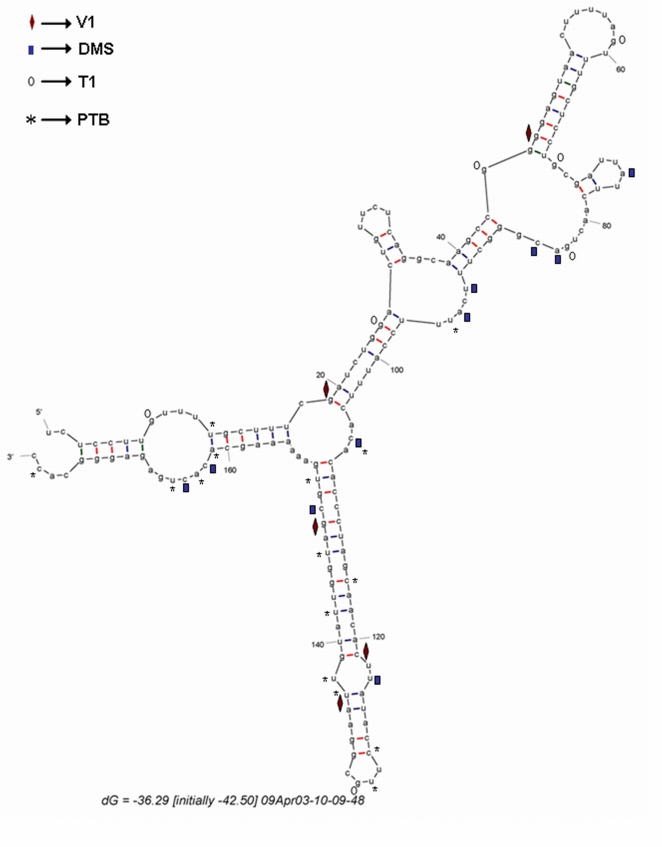
Schematic diagram MFOLD structure of IRF2 5′UTR: Predicted secondary structure of IRF2 5′UTR generated by MFOLD indicating putative contact points of PTB, DMS modification, RNase V1 or T1 cleavage sites.

The nucleotide modifications (by DMS), the pauses corresponding to nuclease (TI and VI) cleavage sites and the toe prints corresponding to PTB binding sites were mapped to MFOLD predicted structure for clarity (Fig. 3). The results suggest that the IRF2 5′UTR contains distinct stem-loop structures and PTB binds to multiple points within the 5′UTR.

### 3′ deletion mutant of IRF2 5′ UTR RNA showed reduced PTB binding

**Figure 4:**
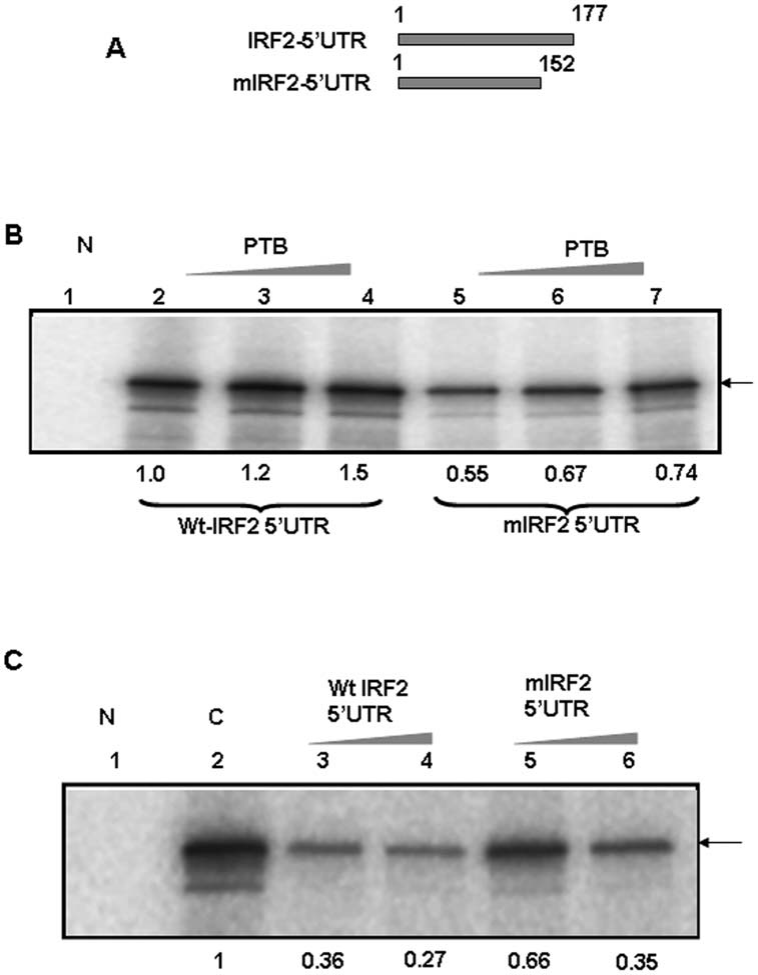
UV Crosslinking of PTB with wt IRF2 5′UTR and 3′ end deletion mutant: (A) A schematic representation of wt and mIRF2 UTRs used in UV-cross linking and translation studies. (B) Purified PTB was incubated with ^32^PIRF2 5′UTR (lanes 2–4) or with ^32^P mIRF2 5′UTR RNA (lanes 5–7). The RNA protein complexes were UV cross linked and analyzed on SDS 10% PAGE followed by phosphorimaging. Lane N represents no protein control. (C) Purified recombinant PTB protein was incubated with ^32^P IRF2 5′UTR in absence (lane 2) or presence of 100 and 200 fold molar excess of self cold IRF2 5′UTR (Lanes 3–4) or mIRF2 RNA (lanes 5–6). The RNA protein complexes were UV cross linked and analyzed on SDS 10% PAGE followed by phosphorimaging. Lane N represents no protein control. The bands were quantified using densitometric analysis and the values have been represented below the panel.

The toe-printing data showed that the contact points of PTB are predominantly at the 3′ end of the IRF2 5′UTR RNA. To investigate this further, 25 nucleotides were deleted at the 3′end from the IRF2 5′ UTR to generate mIRF2 (Fig. 4A). To compare the PTB binding ability of the wt and mutant IRF2 IRES, UV cross-linking experiment was performed using increasing concentrations of the purified PTB protein. As expected, the mutant RNA showed lesser binding as compared to the wild type RNA (Fig. 4B). To validate this further, competition UV cross-linking experiment was performed using unlabeled wt and mIRF2 UTR RNAs. For this purpose ^32^P labeled wild type IRF2 5′UTR RNA was incubated with 0.87 nM of purified PTB protein in absence and presence of increasing concentrations of the competitor RNAs. Results showed that 100 and 300 fold molar excess of the cold self IRF2 5′UTR RNA successfully competed PTB binding with the radio labeled 5′UTR probe (Fig. 4C), whereas the mutant IRF2 RNA showed less efficient competition compared to the wild type (Fig. 4C).

### 3′ deletion mutant of IRF2 5′ UTR RNA show reduced IRES activity

**Figure 5:**
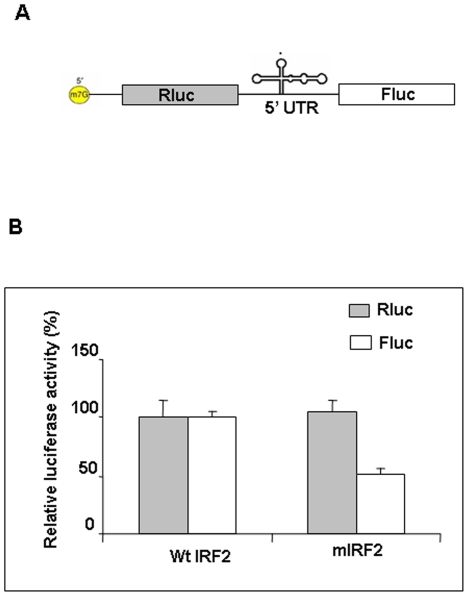
Deletion of 3′ end IRF2 UTR RNA showed reduced activity: (A) Schematic representation of bicistronic construct used for translation studies. (B) Bicistronic plasmid (1 µg) either pRΔEIRF2F or pRΔE3′δ25IRF2F were transiently transfected into HeLa cells. 16 hrs post transfection, respective luciferase activities corresponding to Fluc and RLuc were measured and shown separately. Transfection efficiencies were normalized by co-transfecting with a β-galactosidase plasmid. The data represents mean±s.d. from three independent experiments.

Since 3′ deletion mutant showed reduced PTB binding, we hypothesized that this might also affect the IRF2-IRES activity. To investigate it, the deletion mutant was cloned in the bicistronic plasmid construct (Fig. 5A). Both wild type and mutant bicistronic plasmids (pRΔEIRF2F, pRΔEmIRF2F) were transfected in HeLa cells. The upstream reporter (Renilla luciferase) of the bicistronic RNA was translated by cap-dependent mode, whereas the downstream reporter (Firefly luciferase) was translated by the IRES element. The results showed considerable decrease in firefly luciferase activity in the mutant (pRΔEmIRF2F), suggesting that loss of PTB binding might have direct consequence with the IRES activity (Fig. 5B).

### PTB binding to IRF2 IRES triggers conformation alteration

**Figure 6:**
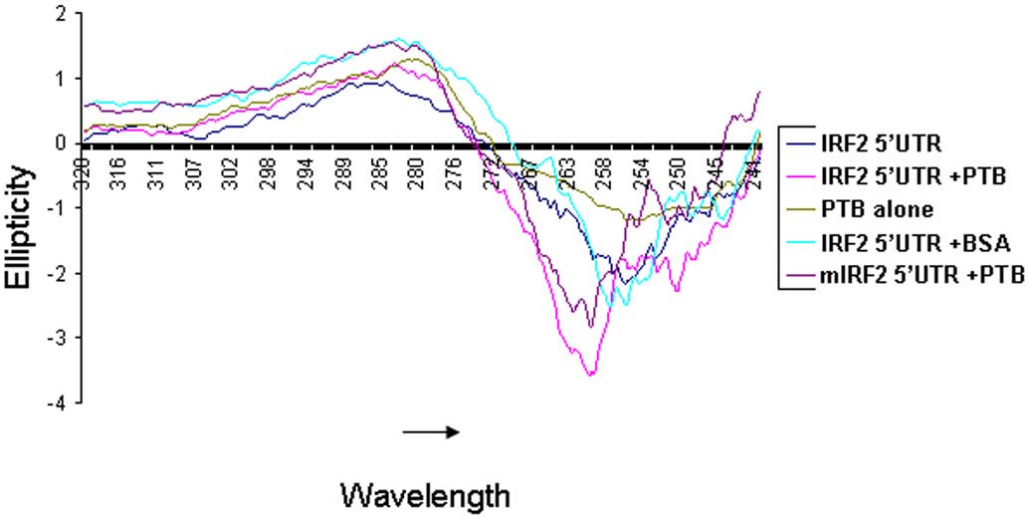
CD spectroscopic analysis of IRF2 in presence of PTB protein: CD spectra were obtained in 0.5 ml RNA-binding buffer between 240 and 320 nm wavelength range at 20°C with IRF2 5′UTR or mIRF2 5′UTR in absence or presence of purified PTB or bovine serum albumin (BSA).

To further verify the possible conformational change in the RNA upon protein binding CD spectroscopic studies were performed. CD spectra of IRF2 5′UTR RNA was analyzed in absence and presence of purified recombinant PTB protein. CD spectra were obtained in 240 to 320 nm range at 20°C in 0.5 ml RNA binding buffer. The results showed distinct change in the wavelengths at which the peak of ellipticity value is reached for RNA incubated with PTB (260 nm) compared to RNA alone (256 nm). Also, at this particular wavelength there was significant increase in the ellipticity value suggesting a change in the conformation of the IRF2 5′UTR RNA in presence of PTB (Fig. 6). Interestingly, CD spectra of mutant RNA with PTB showed less change in the ellipticity value as compared to the wild type RNA (Fig. 6). As a negative control, BSA was incubated with the IRF2 RNA, which did not show any change in the ellipticity value of the RNA. A CD spectrum of PTB alone was performed and has been depicted in figure 6. Taken together the results suggest possible role of PTB protein in mediating conformational alteration in the IRF2 IRES structure to facilitate internal initiation of translation.

### PTB depletion in S10 extract lead to reduced binding of other *trans* acting factors

**Figure 7:**
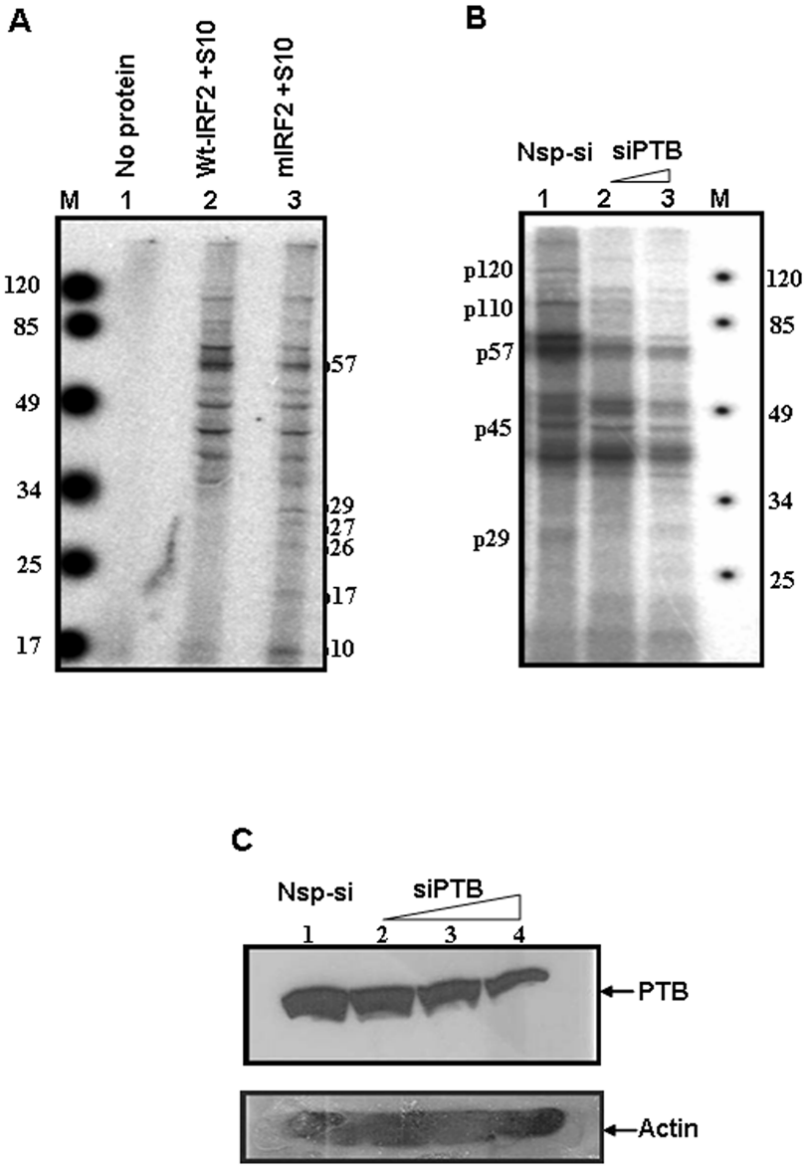
PTB protein helps interaction of other transacting factors with the IRF2-IRES: (A) ^3^ ^2^P labeled IRF2 WT 5′UTR and mIRF2 5′UTR were crosslinked with total HeLa S10 extracts and run on an 10% SDS PAGE followed by phosphorimaging. Lane 1 represents no protein control. Lane M indicates the molecular mass marker. (B) 70% confluent HeLa cells were transfected with 60 nm and 80 nm of siPTB or nsp si as indicated. 36 hrs post-transfection the cells were harvested and subjected for western blot analysis to check PTB levels. S10 extract was prepared from these cells and UV Crosslinking was performed as mentioned previously. Lane M indicates the molecular mass marker. (C) Western-blot analysis showing the reduced levels of PTB with increasing concentration of siPTB. Actin protein was detected as loading control.

We have also investigated the binding profile of HeLa cell extracts with IRF2 UTR. ^32^P labeled Wt and mIRF2 UTR RNAs were incubated with the extracted total proteins, UV-crosslinked and analysed on SDS-PAGE. The results indicated that wtIRF2 5′UTR interacted strongly with 57 kDa protein (PTB), whereas with mIRF2 5′UTR the intensity of the band corresponding to 57 kDa protein was relatively less. Interestingly, the mIRF2 showed binding with additional proteins, some of those could be the cleavage products of PTB (p25), which might negatively influence IRES activity [21] (Fig. 7A). Finally to investigate whether PTB facilitates interaction of other *trans* acting factors to enhance translation, PTB depleted HeLa S10 was used in UV cross linking study. PTB was partially silenced in HeLa cells by using increasing concentration of siPTB. 36 hours post transfection S10 extracts were prepared. A significant decrease of PTB protein level was observed at 60 nm & 80 nm concentration of siPTB. UV-crosslinking experiment was performed using wt IRF2 RNA probes and S10 extracts from control or siPTB treated cells. Interactions of several cellular proteins were found to be significantly reduced (Fig. 7B). These include proteins of range approximately p120, p110, p57, p45, p29. Additionally, the extract was subjected for western blot analysis to confirm PTB silencing (Fig. 7C). The results lead us to hypothesize that PTB might play a crucial role in bringing some of these proteins to facilitate ribosome loading and enhance translation initiation.

### PTB enhances ribosome assembly at the IRF2 IRES

**Figure 8:**
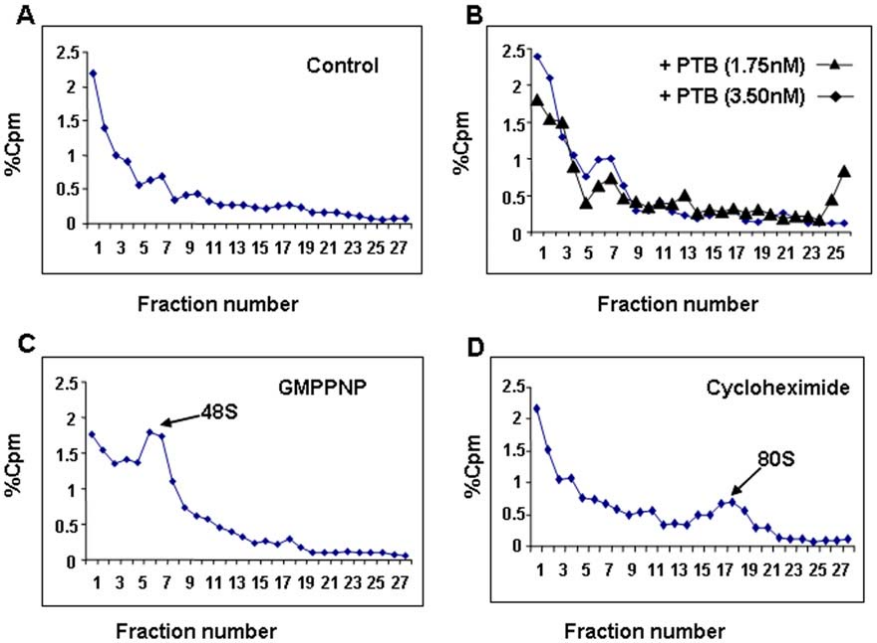
Analysis of translation initiation complex formation in presence of recombinant PTB: ^32^P UTP labeled IRF2 UTR was incubated with RRL and amino acid mix and analyzed by sucrose density gradients. Panel A represents the control. Panel B shows the profile of the respective reactions supplemented with either 1.75 or 3.5 nM of purified recombinant PTB protein (as indicated). The panels C–D shows the effect of addition of either GMPPNP (panel C) or Cycloheximide (panel D) used to confirm the 48S and 80S ribosomal peaks (indicated by arrows).

Further we wanted to study the effect of PTB on IRF2 IRES mediated translation initiation complex formation. For this purpose, ^32^P UTP labeled IRF2 UTR RNA was incubated with in the *in vitro* translation mix containing RRL and amino acids. The reactions were supplemented with purified PTB in two reactions. The ribosome complexes were resolved on sucrose density gradients as described in materials and method. GMPPNP (which terminates ribosome assembly at the 48S stage) and cycloheximide (which trap 80S ribosomal complexes) were used to confirm the 48S and 80S ribosomal peaks. The results showed that the supplementation of PTB marginally enhanced the formation of 48S initiation complex compared to control (Fig. 8). It is possible that PTB binds to IRF2 UTR and melts the secondary structure to facilitate the landing of 43S ribosome on to the IRF2 IRES.

## Discussion

The mechanism of IRES mediated translation in cellular RNA is still not completely understood. Various IRES *trans* acting factors or ITAFs have been implicated to have role in mediating internal initiation of translation. Polypyrimidine-tract-binding protein (PTB) has been implicated as an important ITAF for a number of viral as well as cellular IRESs. It is believed that PTB has a chaperone like activity that can bring about a change in the conformation of the IRES structure, which might facilitate the interaction of the ribosomes with the RNA leading to efficiently initiate translation.

In this paper, we have investigated the interaction of PTB with the IRF2-IRES RNA and studied its importance on its translation initiation. Multiple contact points were mapped on the IRF2 IRES RNA. A significant number of those contact points were mapped to the 3′ end of the IRES element. In fact, Apaf-1 IRES is also known to have two potential PTB binding sites at the 3′ end region [13]. This might be a logistic position for PTB binding to facilitate binding of the ribosome in the vicinity of the iAUG, the translation start site. Deletion of 25 nt from the 3′end of the IRF2 IRES RNA showed reduced PTB binding. Although, the IRES function was severely impaired, it was not completely abrogated perhaps due to residual PTB binding through other sites within the 5′UTR, which might still contribute to IRES function. It is also possible that there is a minor change in the local structure of the mutant RNA due to deletion that has contributed to the decrease in the IRES activity.

However recent report suggests that the IRES activity might not completely depend on the overall structure but on different sequence modules that play important role in the ITAF recruitment [19]. This was supported by UV crosslinking for Wt and mIRF2 RNA probes in presence of purified PTB and HeLa S10 extracts. As expected mIRF2 showed significantly reduced binding with PTB when compared to wtIRF2. Also, in HeLa S10 extracts, we found that the wt IRF2 RNA showed a stronger binding with the 57 kDa protein as compared to the mIRF2. Interestingly, smaller polypeptides were found to specifically bind to the mIRF2. At this stage we are not sure about the functional significance of the binding of these smaller polypeptides to the mIRF2; however it is tempting to speculate that these proteins might negatively influence the translation initiation in the mIRF2 RNA.

Nuclease mapping and chemical modifications partly revealed the secondary structure of the IRF2 5′UTR, which largely supports the MFOLD prediction. DMS modification interference assay indicated perturbation of the RNA structure in presence of PTB. The presence of PTB masked the RNA, rendering it inaccessible to DMS modifications at certain places. Also, a few new DMS modification sites were found under such condition, suggesting changes in local structure due to protein binding. CD spectroscopic analysis reinforces the idea of change in RNA conformation upon protein binding. In fact when CD experiment was performed with the mutant IRF2 IRES RNA and the PTB protein, change in molar ellipticity was found to be less than that obtained with the wild type RNA. PTB has been shown to have chaperone like activity, thus it would be interesting to study how its interaction with the IRF2 5′ UTR RNA and consequent change in the RNA conformation helps in ribosome assembly during internal initiation. Also it is possible that such a change in the conformation of the RNA might recruit certain other *trans* acting factors near the vicinity of the translation start site that would facilitate the recruitment of the ribosomes to the RNA and thus facilitating translation initiation. To further consolidate the observation, S10 extract isolated from HeLa cells, partially silenced for PTB expression was used for binding using Wt IRF2 probe. Several cellular *tran-*acting factors were unable to bind Wt IRF2 in reduced presence of PTB. Interestingly, we found that supplementing the *in vitro* translation mix with purified PTB marginally enhanced the formation of the 48S ribosomal initiation complex.

Thus we hypothesize that PTB serves as an ITAF which recruits other possible *trans*-acting factors that could facilitate IRES mode of translation initiation in the IRF2 mRNA. However, at this stage it is not clear how PTB or other IRES *trans*-acting factors (ITAFs) interaction helps in switching on the IRES activity of the cellular IRES elements. It could be a complex interplay of PTB and several other ITAFs. Future experiments would be directed to understand how the ITAFs binding directs the ribosome to land preferentially to the IRES site and translate by internal initiation under stress conditions.

## Materials and Methods

### Ethics Statement

No, an ethics statement is not required for this work.

### Plasmid constructs

The bicistronic constructs containing respective 5′UTR sequences of full length IRF2 (NCBI accession number NM_002199) and 3′ deletion IRF2 (pRΔEIRF2F, pRΔEmIRF2F) were cloned downstream of the landscape of structure derived form the inactive part of ΔEMCV IRES sequence between Renilla luciferase (RLuc) and Firefly luciferase (FLuc) genes, in HindIII and EcoRI sites. The construct pRΔEnullF [20] was a kind gift from Dr. Peter Sarnow (Stanford University). For constructing IRF2 monocistronic plasmid pIRF2Fluc and mIRF2Luc were generated from respective bicistronic plasmids by releasing out the insert IRF2 Fluc by HindIII and ApaI enzymes (NEB) and cloned in pCDNA 3.1- Fluc.

### Cell lines and Transfection

HeLa S3 cells were maintained in DMEM (Invitrogen) with 10% fetal bovine serum (GIBCO, Invitrogen). Cells were transfected with various bicistronic plasmids and pSV40β-gal using Tfx 20 reagent (Promega) and luciferase assay was performed using Dual luciferase assay reagent (Promega). Transfection of siRNA was performed in HeLa S3 cells growing in monolayer using Lipofectamine-2000 transfection reagent and optiMEM-I prepared without addition of antibiotic (Invitrogen). Cells were seeded onto 100 mm dishes in duplicates one day prior to transfection in similar manner. Transfections were performed with 40 nm, 60 nm and 80 nm of siPTB (Dharmacon) or 80 nm of nonspecific si (Dharmacon) diluted with optiMEM-I to a final volume of 100 µl followed by incubation at room temperature for 20 minutes. Subsequently, 4.9 ml of optiMEM-I was added to the transfection mixture, which was then layered onto cells. 4 hours later the medium was replaced with 9 ml of DMEM (with antibiotic) and 10% FBS. 36 hours post transfection the cells were washed with PBS, and were trypsinized followed by HeLa S10 extract preparation. S10 extract was prepared from HeLa cells as described before (22).

### *In vitro* Transcription

The IRF2 5′UTR and mIRF2 RNA probes were made from IRF2Fluc and mIRF2Luc DNA plasmids respectively linearized with Eco RI and was transcribed by T7 RNA polymerase. Similarly, the ^32^P labeled RNA probes corresponding to the 5′UTRs of IRF2 and deletion mutant IRF2 were made from their respective plasmid DNAs after linearizing with EcoRI and transcribed with T7 RNA polymerase and 10 µCi/µl of alpha ^32^P UTP (NEN) as per manufacturer's guidelines.

### Purification of recombinant PTB

The expression of recombinant PTB (NCBI accession number NM_031990) from PET28a-PTB (a generous gift from Dr. J.G. Patton) was induced by 0.6 mM IPTG in *E.coli* (BL21 DE3) cells transformed with the expression vector. His-tagged protein was purified using Ni^2+^-nitrilotriacetic acid-agarose (Qiagen) under non-denaturing conditions and eluted with 250 mM imidazole.

### Ribonuclease probing and DMS modification

10 µg of *in vitro* transcribed IRF2 bicistronic RNAs were denatured at 65*°C* for 10 minutes and then cooled slowly to room temperature. The RNAs were then digested with 0.5 unit of RNase T1 (SIGMA) for 15 minutes at 37°C. After incubation the reaction mixture was treated with proteinase K followed by phenol:chloroform extraction and alcohol precipitation. For RNase V1 (Ambion) digestion, the RNA was incubated with 0.01 units of RNase V1 for 10 minutes at 30°C. After the incubation the reaction mixture was extracted with phenol: chloroform and alcohol precipitated. T1 digestions were carried out in the buffer containing 10 mM Tris.Cl (pH 7.5), 10 mM MgCl_2_, and 100 mM KCl. The DMS modification was carried out in the buffer containing 50 mM sodium cacodylate (pH 7.5), 5 mM MgCl_2_, 100 mM KCl at 30°C for 10 minutes using 1 µl of DMS diluted 1∶8 in absolute ethanol. The modified RNAs were precipitated with ethanol. The digested and the modified RNAs were reverse transcribed using AMV RT (Promega) with an end labeled primer annealing to 1–20 at the 5′ end of the firefly luciferase gene. The cDNAs were precipitated with 0.3 M sodium acetate and absolute ethanol and then resolved in a 8M urea-8% Acrylamide gel electrophoresis along with a reference sequencing reaction (fmol kit, Promega) using the same end-labeled primer. Similarly, purified PTB bound RNA in buffer was modified with 1 µl of DMS diluted 1∶8 in absolute ethanol at 30°C for 10 minutes The modified RNAs were precipitated with ethanol the modified RNAs were reverse transcribed using AMV RT (Promega) with an end labeled primer annealing to 1–20 at the 5′ end of the firefly luciferase gene and was analyzed as mentioned before.

### Primer extension inhibition analysis or Toe-printing

Increasing concentration of purified his-tagged PTB protein (500 and 800 ng) was incubated with 5 pmoles of *in vitro* transcribed RNA corresponding to the IRF2 bicistronic RNA (derived from the construct pRIRF2F) and binding reaction was performed in a final volume of 20 µl at 30°C for 20 minutes. Reaction where PTB was not added serve as a negative control. To the reactions, [^32^P]-end labeled primer complimentary to 20 nucleotides of the 5′ end of the Firefly luciferase was added and allowed to extend using 3 units of AMV-Reverse transcriptase (Promega) at 30°C for one hour. The cDNAs were alcohol precipitated, resuspended and compared with the dideoxynucleotide sequence ladders (obtained using same end-labeled primer) by electrophoresis on a 6% polyacrylamide/8M urea denaturing gel.

### CD spectroscopy

Measurements of CD spectra were performed with a Jasco J-715 spectropolarimeter. Spectra were obtained in 0.5 ml RNA-binding buffer (5 mM HEPES, pH 7.6, 25 mM KCl, 2 mM MgCl_2_, 3.8% glycerol, 2 mM DTT, 0.1 mM EDTA). CD spectra were obtained in 240 to 320 nm range at 20°C with IRF2 5′UTR (400 ng) and 800 ng of PTB or bovine serum albumin (BSA).

### UV-cross linking

RNA binding and UV crosslinking experiment was carried out following the details described earlier (5). Briefly, [α-^32^P] 5′UTR RNAs were allowed to form complex with purified PTB or the cytoplasmic extract followed by cross-linking with UV light. The unbound RNAs were digested with RNase A treatment. The protein RNA complexes were then resolved in a SDS-10% polyacrylamide gel followed by phosphorimaging analysis.

### Western-blot analysis

Protein concentration of the S10 extract prepared from HeLa cells were assayed by Bradford (Bio-Rad). Equal amounts of cell extracts were loaded on SDS-10% polyacrylamide gel. The resolved proteins were electro-transferred to nitrocellulose membranes. Samples were then analysed by mouse monoclonal anti-PTB antibody (Calbiochem), followed by secondary antibody (horseradish peroxidase- conjugated anti-mouse; Sigma). Rabbit polyclonal anti-actin (Sigma) was used as control for equal loading of total cell extracts. Antibody complexes were detected using the enhanced chemiluminescence (ECL) detection kit (Amersham Antibodies).

### Ribosome assembly

IRF2 UTR RNA was labeled with ^32^P UTP and the 25 µl reactions containing 17.5 µl RRL (Promega), 1.5 µl of amino acid mix (Promega) and PTB was supplemented as indicated in the figure legends. Labeled RNA 100,000 cpm was used per reaction. The reactions were incubated at 30°C for 20 min. and the volume was made upto 150 µl with the sucrose density gradient buffer (20 mM Tris HCl, pH 7.5, 100 mM KCl, 2 mM MgCl_2_ and 1 mM DTT). The reactions were overlaid on 5–30% linear sucrose density gradients and centrifuged for 3 hrs at 30,000 rpm using SW41 rotor in Beckman ultracentrifuge. 200 µl fractions were collected from top of the radioactive tube. Counts of each fraction were measured and %cpm was plotted against the fraction number.

## Acknowledgments

We gratefully acknowledge members of our laboratory for their help and suggestions.
